# Prevalence of abnormal uterine bleeding in Brazilian women: Association between self-perception and objective parameters

**DOI:** 10.1371/journal.pone.0282605

**Published:** 2023-03-13

**Authors:** Gabriela Pravatta Rezende, Daniela Angerame Yela Gomes, Cristina Laguna Benetti-Pinto

**Affiliations:** Department of Obstetrics and Gynecology, School of Medical Sciences, University of Campinas, Campinas, São Paulo, Brazil; Teikyo University, School of Medicine, JAPAN

## Abstract

**Introduction:**

Abnormal uterine bleeding (AUB) is the main cause of demand for gynecological care during the reproductive period, with negative consequences on women’s lives. In Brazil, data on the prevalence of AUB is scarce and does not reflect the national reality.

**Objective:**

To evaluate the prevalence of AUB and associated factors in Brazil.

**Methods:**

Multicenter cross-sectional study, including 8 centers representing the 5 official geographic regions of Brazil. It included postmenarchal women who answered a sociodemographic questionnaire, with socioeconomic stratum and data related to uterine bleeding (self-perception of AUB and objective data)

**Results:**

1928 women were included, with 35.5±12.5 years of age, 167 postmenopausal. The 1761 women in their reproductive period, had a menstrual cycle duration of 29.2±20.6 days, with bleeding for 5.6±4.0 days. In these, the prevalence of AUB, considering self-perception by the women, was 31.4%. Only among women who considered their bleeding abnormal, the menstrual cycle lasted less than 24 days in 28.4%, bleeding lasted longer than 8 days in 21.8%, 34.1% reported intermenstrual bleeding and 12.8% reported postcoital bleeding. Also, regarding these women, 47% reported a previous diagnosis of anemia, with 6% requiring intravenous treatment (iron or blood transfusion). Half of the women mentioned that the menstrual period had a negative impact on quality of life, while this worsening occurs in about 80% of those with self-perception of AUB.

**Conclusion:**

In Brazil, the prevalence of AUB is 31.4%, assessed by self-perception, in agreement with objective AUB parameters. The menstrual period has a negative impact on the quality of life of 8 out of 10 women with AUB.

## Introduction

Abnormal uterine bleeding (AUB) is the term used for changes in blood flow from the uterine body, characterized as abnormal in regularity, volume, frequency, or duration, in the absence of pregnancy [1, 2]. It may also represent excess menstrual loss that occurs in isolation or in combination with other symptoms, including social, material, emotional, physical repercussions, and impairments in quality of life [3]. Complaints related to menstrual disorders are the leading cause of gynecological care. In addition, AUB accounts for two-thirds of hysterectomy indications around the world and is the most common cause of iron deficiency anemia in women during their reproductive period [4, 5].

Women with AUB have higher rates of absenteeism at school and loss of workdays, due to associated symptoms or possible episodes of social embarrassment, mainly related to the fear of blood leaking to the clothes, causing reduced productivity and financial consequences [5–7]. Data in the United States (USA) shows that spending on the treatment of menstrual disorders exceeds US$1 billion per year and more than US$12 billion is spent to meet the drop in income related to abnormal bleeding [8]. In Brazil, only in January 2022 approximately 2.768 abdominal hysterectomies were performed, 2/3 of them due to AUB [9].

Data from world literature shows that the prevalence of AUB ranges between 5 and 60%, depending on the population evaluated [10–17]. Considering two large countries, in the USA it is estimated that 5.3% of women aged between 18 and 50 years have some type of menstrual alteration [10]; while in China, a cross-sectional study showed a 60.8% prevalence of AUB [11]. In Brazil, a country with continental dimensions, with political, social, cultural, and economic differences between regions, the data on the prevalence of AUB is scarce and does not allow any reflections on the reality of the country. In 2013, a study conducted in the south of the country showed a 46.4% prevalence of AUB, with excessive bleeding being the most frequent complaint (23.2%) [18]. In the Western Amazon, in northern Brazil, a retrospective analysis of women seen during 3 years in gynecology outpatient clinics showed that AUB was the cause of 22.1% of total care, with menstrual flow as the main complaint in 91% of these women [19]. Both studies are regional. The population of Brazil is about 214 million people, of which 51.7% are women [20]. This study was developed to evaluate the prevalence of AUB with representativeness throughout the country, evaluating self-perception, complaints, and repercussions of the menstrual period, to provide data that can help in the implementation of public policies, improving knowledge and care for this population.

## Methods

A cross-sectional population-based, descriptive, multicenter study, including 8 representative centers from the five official geographic regions of Brazil, including women who had already reached menarche, randomly recruited in outpatient medical consultations, not specialized in taking care of abnormal bleeding. Pregnant women, lactating women, with a history of hysterectomy or cognitive difficulty that prevented them from understanding the questions asked were excluded.

All of them answered a questionnaire containing sociodemographic data, socioeconomic stratum assessment (according to official criteria and defined by the Brazilian Association of Research Companies, ABEP) [21], personal history and data related to uterine bleeding, such as age of menarche, history of anemia and hemotransfusion due to AUB, self-perception of menstruation (self-perception of AUB), menstrual pattern in relation to regularity, volume, frequency or duration, use of sanitary products for menstrual blood collection, need to change clothes due to blood leaking, knowledge of AUB etiology, impact on quality of life and difficulty in accessing health services for treatment of AUB (using the parameters of the Visual Analog Scale—EVA where zero means no impact/no difficulty and ten is the worst possible impact/extreme difficulty) [22]. Comparison to the normality parameters defined by FIGO [6] was used for the analysis of the parameters and limits associated with the menstrual period.

The proportion estimation formula was used to calculate the sample size in a descriptive study with a categorical qualitative variable. To estimate the prevalence of AUB, studies from literature [18, 19] were used, setting the level of significance alpha or type I error at 5% (alpha = 0.05) (or 95% confidence interval) and the sampling error at a few values (3.0%, 4.0% and 5.0%). Considering a prevalence of 46.4% and a sampling error of 3.0%, a minimum sample of n = 1760 participants was obtained, divided into at least 220 women per center. The computational program—SAS System for Windows (Statistical Analysis System), version 9.4, was used for the statistical analysis. SAS Institute Inc, 2002–2012, Cary, NC, USA. Continuous variables were described as mean and standard deviation or median/minimum and maximum value and evaluated through the Intercooled Stata 13.0 program. Chi-Square or Fisher’s Exact Test and Mann-Whitney’s nonparametric text were used to compare categorical variables.

This research was approved by the ethics committee of the coordinating institution (CAAE 40654720.0.1001.5404) and by the ethics committee of each participating center. All women were invited to participate voluntarily and signed the Informed Consent Form prior to their inclusion. When necessary due to age, a written assent form was applied and a consent form was obtained from parents or guardians. This was an investigator-initiated study with financial support from Bayer S.A. The funding sources had no role in the study conduct, analysis, interpretation of data, or decision to publish the results. The study was carried out with the support of the Brazilian Federation of Gynecology and Obstetrics (FEBRASGO).

## Results

A total of 1,928 women were included from the five different geographic regions of Brazil, 772 from the Southeast (40%), 460 from the Northeast (24%), 240 from the South (12%), 230 from the North (12%) and 226 from the Midwest (12%). The mean age and BMI of the women was 35.54 ± 12.48 years and 25.39 ± 5.02 kg/m^2^, respectively, and 10.1% (n = 195) were less than 20 years old, 50.8% (n = 980) between 20–39 years of age, 26.1% (n = 504) 40–49 years of age and 1 2.9% (n = 249) were aged 50 years or older (Table 1). Although it was not the objective of this study, it was found that 56% of the women were overweight or obese (BMI≥ 25 kg/m^2^). The predominant ethnicity was white (about 60%) and brown (31.2%).

**Table 1:** Sociodemographic characteristics of the total sample of women (n = 1928) and women in reproductive periods (n = 1761).

	Total sample of women (n = 1928)	Women in reproductive period (n = 1761)
Variable	Average ± SD or n (%)	Average ± SD or n (%)
Age	35.54 ± 12.48	33.40 ± 10.55
<20 years of age	195 (10.10%)	195 (11.07%)
20–39 years of age	980 (51.00%)	978 (55.54%)
40–49 years of age	504 (26.00%)	489 (27.77%)
> 50 years of age	249 (12.90%)	99 (5.62%)
Ethnicity		
White	1147 (59,49%)	1036 (58,83%)
Non-white	781 (40.6%)	725 (41,17%)
BMI	25.39 ± 5.02	25.25 ± 5.07
Complete years of study	14.44 ± 5.65	14.47 ± 0.70
Social Stratification		
Class A	344 (17.85%)	367 (20.85%)
Class B1	263 (13.64%)	273 (15.50%)
Class B2	402 (20.85%)	404 (22.94%)
Class C1	354 (18.36%)	260 (14.76%)
Class C2	336 (17.42%)	251 (14.25%)
Class D/E	229 (11.88%)	206 (11.70%)
Age of Menarche	12.44 ± 1.54	12.39 ± 0.70
Number of pregnancies	1.31 ± 1.60	1.19 ± 0.70
Menopause	167 (8.66%)	—

Among the total number of women, 167 women declared they were in the postmenopausal period (8.66% of the total), and only 1 of them reported an isolated episode of postmenopausal bleeding (abnormal postmenopausal bleeding of 0.60%). Thus, 1,761 women in their reproductive period were included, for whom the characteristics of the menstrual cycle and the prevalence of AUB were evaluated. For these, the mean age of menarche was 12.4±0.7 years and the average number of pregnancies was 1.19±0.70. The majority (63%) participated economically in family support, and 22% of them declared themselves as being the only ones financially responsible for the family. The average number of complete years of study was 14.4±0.7; 21% were socioeconomically classified as class A; 15% as B1; 23% as B2; 15% as C1; 14% as C2 and 12% as D-E classes. The prevalence of AUB, considering female self-perception, was 31.4% (n = 553). The prevalence of anemia secondary to abnormal bleeding was 47.0% and, among these, 6.3% required intravenous treatment with iron or blood transfusion (2.2%). Considering only women with AUB, 41.7% (n = 231) of them reported having undergone some treatment to reduce blood loss, the most frequent being the use of combined oral contraceptives, used by 35,9% of them, followed by the levonorgestrel intrauterine system and isolated progestogens. Only 6 women in this group reported having already undergone conservative surgical procedures to control AUB.

Regarding the characteristics of the menstrual cycle, using normality criteria defined by FIGO [6], it was found that among all women in their reproductive period, the mean duration of the menstrual cycle was 29.2±20.7 days, with menstrual bleeding for 5.6±4.0 days. Considering the bleeding parameters, the menstrual cycle lasted less than 24 days in 18.3%, menstrual flow lasted longer than 8 days in 8% and there was a combination of these alterations in 3.6% of them. Intermenstrual bleeding and postcoital bleeding were reported by 16.5% and 7.0% of them, respectively, while cycles were long or infrequent in 7.8% of women (Table 2). In addition, 22.5% of them reported the need for simultaneous use of more than one type of sanitary product to contain the bleeding. Divided by age group, it was found that, after 50 years, 48% of them reported self-perception of AUB. In the other age ranges, in order of prevalence, self-perception of AUB was observed in 40.5% of women aged 40–49 years, 27.2% between 20–39 years of age and 20.5% under 20 years of age.

**Table 2:** Data on bleeding in women in reproductive period (n = 1761) and in women with self-perception of normal bleeding or with AUB.

Bleeding parameters	Total sample	Normal Menstruation (N = 1208)	Abnormal Menstruation (AUB) (N = 553)
Interval between periods (days)	29.2 ± 20.7	27.22 ± 10.21	33.6 ± 33.2
Duration of menstrual flow (days)	5.6 ± 4.0	4.82 ± 1.59	7.3 ± 6.2
Pads used per cycle (number)			
< 8	414 (24.34%)	1085 (89.82%)	93 (16.82%)
8–16	884 (51.97%)	82 (6.79%)	213 (38.51%)
>16	403 (23.69%)	41 (3.39%)	247 (44.67%)
Intermenstrual bleeding (n %)	291 (16.51%)	102 (8,4%)	189 (34,1%)
Postcoital bleeding	124 (7.04%)	52 (4,3%)	71 (12,8%)
Impact of bleeding in quality of life (0–10) (mean ± SD)	5.22 ± 3.13	4.50 ± 2.94	6.82 ±2.91
Difficulty in care (0–10)	3.40 ± 3.21	3.2 ± 3.5	4.3 ± 6.4

**Fig 1:**
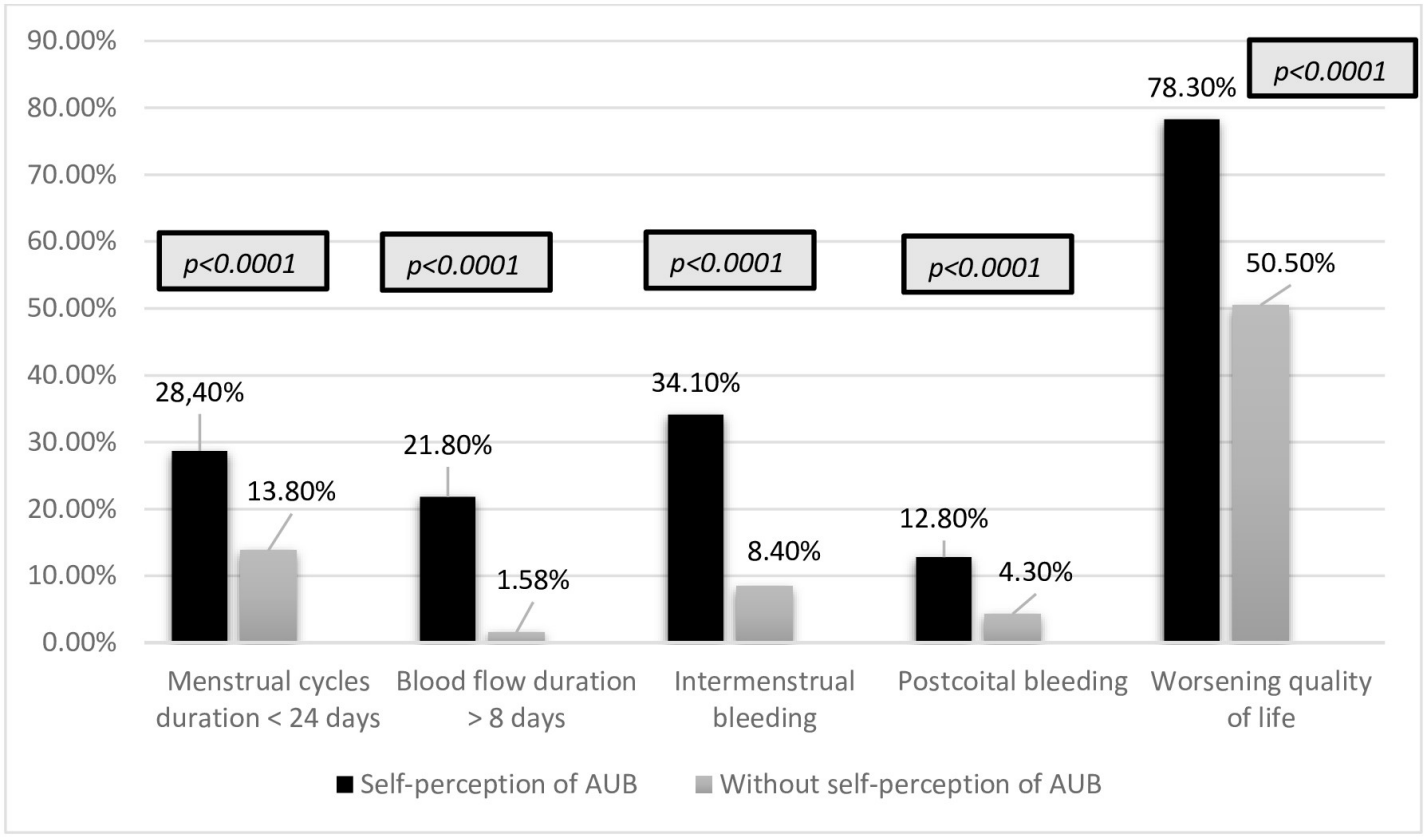
Comparison of women in menacme with (n = 553) and without (n = 1208) self-perception of AUB and objective parameters of AUB according to the 2018 FIGO definition [6]. * Chi-Squared test.

**Fig 2:**
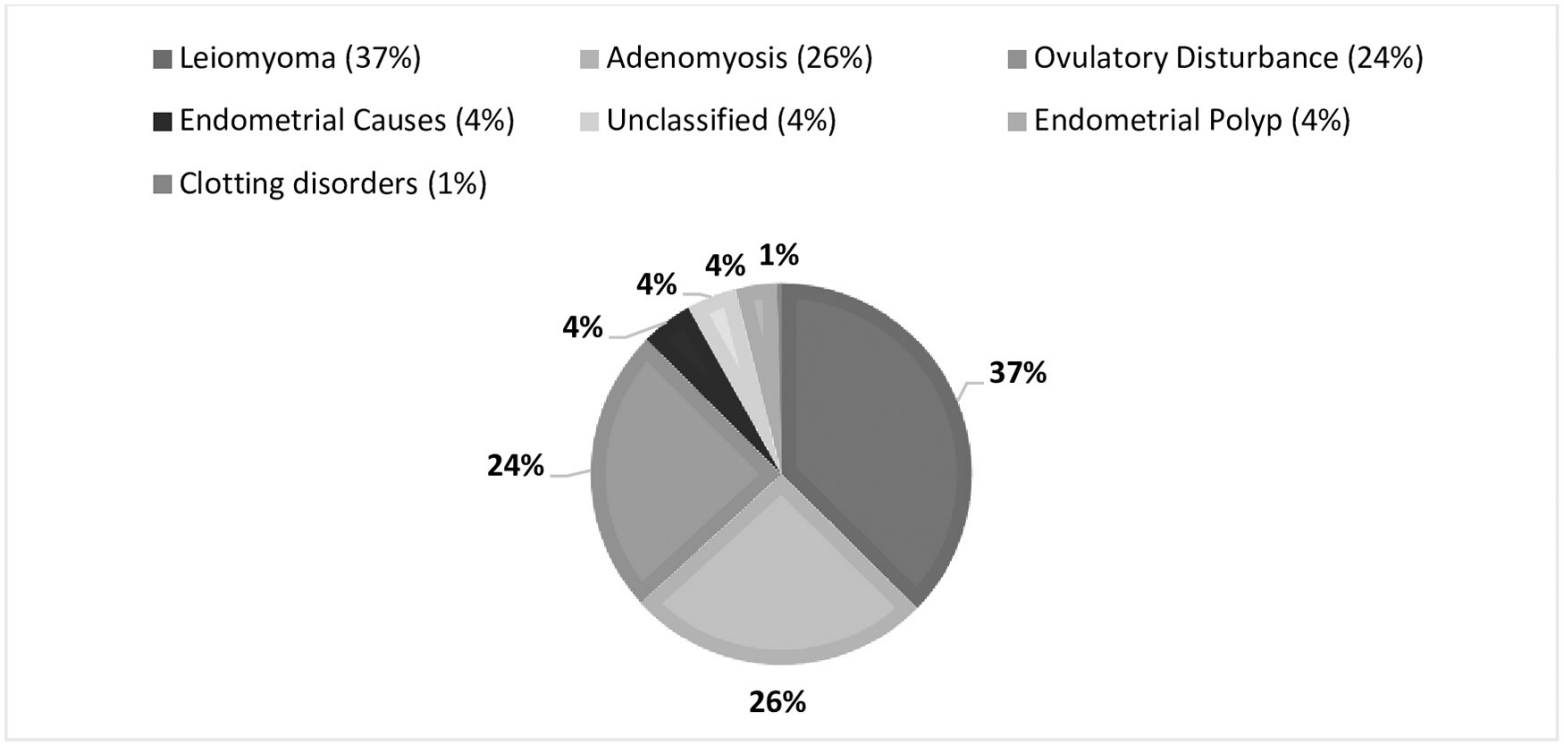
Etiologies of AUB among women with self-perception of AUB and known diagnosis (N = 201).

Regarding only women who considered their bleeding abnormal (self-perception of AUB 31.4%, N = 553), the menstrual cycle lasted less than 24 days in 28.4% (n = 157), menstrual bleeding lasted longer than 8 days in 21.8% (n = 121), while 34.1% (n = 189) reported intermenstrual bleeding and 12.8% (n = 71) reported postcoital bleeding. The interval between menstruations was 33.6 ± 33.2 days and the duration of bleeding was 7.3±6.2 days (Table 2). All objective parameters used to define the presence of AUB were more prevalent among women who declared themselves as having AUB (Fig 1). In the latter, the etiological diagnosis was known by 201 women and distributed as follows: endometrial polyp in 7 (3.48%), adenomyosis in 52 (25.87%), leiomyoma in 75 women (37.31%), coagulopathies in 1 (0.49%), ovulatory causes in 49 (24.37%) and endometrial causes in 8 women (3.98%), i.e., 66% had structural causes of AUB (Fig 2).

When asked if the bleeding period affected their life, about 50% of the women reported that there was some negative impact on their quality of life (mean of 5.2 ± 3.1, considering a scale of 0–10), but this percentage increases to 78.3% among those who considered their bleeding abnormal (self-perception of AUB). It was also found that at least 8 out of 10 women (80.2%) reported at least one other uncomfortable symptom during the bleeding period, the most frequent being dysmenorrhea (66%), mood changes (47%), headache (43%) and changes in bowel habit (28%). Other symptoms reported were lower back pain, body pain, leg pain, weakness and mastalgia, present in 1 to 3% of women.

## Discussion

The prevalence of AUB in Brazil is 31. 4% according to the self-perception of women in their reproductive period. Of these, about 5 out of 10 have already presented anemia due to increased bleeding. Self-perception of AUB was shown to be in accordance with the objective limits established by FIGO for the menstrual cycle, i.e., women who consider their bleeding had increased have menstrual cycles shorter than 24 days or bleed for more than 8 days or still have intermenstrual bleeding and postcoital bleeding more frequently than women who do not consider their bleeding abnormal. Moreover, although the menstrual cycle has a negative impact on quality of life in about half of the women, in those with self-perception of AUB this occurs in 8 out of 10 women. AUB is more prevalent in women in their 40s, being reported by at least 4 out of 10 women. Structural causes, mainly adenomyosis and uterine leiomyoma, represented the main etiological diagnosis of AUB.

Data from literature on the prevalence of AUB is scarce and presents large discrepancies. Thus, a recently conducted cross-sectional study in China demonstrated a 60.8% prevalence of AUB [11]. On the other hand, a large European study, conducted with data from 5 countries (France, Germany, Spain, The Netherlands, and Switzerland), revealed that only 13.1% of the participants reported that their menstrual cycle and blood loss were out of control [12]. In England, the prevalence is 25% [13] and in Scotland, 30–35% of women had complaints of increased menstrual volume, and 20% rated the menstrual period as a "problem" [14]. Iranian data, in turn, shows a 35.8% prevalence of AUB, and changes in menstrual frequency accounted for 23.8% of cases [16]. In India, the prevalence of menstrual disorders affects 18.2% of the female population, with more than 33% of cases secondary to blood flow volume disorders [17].

Our study was conducted in Brazil, a country with continental dimensions; when comparing territorial expansion with other countries where the aforementioned studies were conducted, only the United States and China have larger dimensions. Our data reveals an apparent higher prevalence of AUB in Brazil compared to the USA, but lower when a comparative analysis is performed with China. The differences between the data may be due to the methodology of the studies, but also to differences in social, demographic, and cultural characteristics as well as access to health services. Population-specific studies are an important tool to estimate indicators of health condition, health-related behaviors, and access to health services.

World societies have valued women’s self-perception of bleeding pattern in the diagnosis of AUB. Our data shows that the self-perception of AUB presented agreement with the objective parameters defined by FIGO to classify bleeding as abnormal [6]. Thus, women who considered their menstruation abnormal had shorter cycles, with more days of bleeding, in addition to postcoital bleeding, bleeding outside the menstrual period and worsening of quality of life was significantly higher than in women who did not report abnormalities in their menstrual bleeding. Studies on the approach of AUB by health professionals demonstrate that clinical investigation does not always include parameters valued by women who experience this condition [23]. Regardless of the definition of AUB, the importance of evaluating the woman’s self-perception is currently emphasized, considering the volume of blood loss when the woman refers to it as increased or interfering in her quality of life [6, 24].

The worsening of quality of life during the menstruation period was reported by most of the women evaluated, especially among those with self-assessment of AUB. This can become a social impairment, in personal relationships and work activities. In the United States, a study conducted with African American women revealed that 28% classified their quality of life as regular or poor during the menstrual period and 18% required hospitalization due to AUB [15], with data that more than 12 billion dollars are spent per year to meet the drop in productivity related to menstrual disorders [25]. Considering that 6 out of 10 Brazilian women participated economically in family support and 2 out of 10 were the only ones financially responsible for supporting their family and the recognized association between AUB and absenteeism, it is possible to infer an association with losses in family income and professional income. Almost 30% of women also reported diagnosis and treatment for anemia. Abnormal bleeding alone or when associated with anemia is associated with reduced quality of life in women. There is data showing that up to two-thirds of women with AUB may have anemia [12, 25–27]. Our results were lower, probably reinforcing the difficulty of access to medical care, as reported by the women evaluated. Increasing public health actions for early diagnosis and care as well as reducing health repercussions, may improve some social and economic indicators associated with AUB.

As strengths, this study is the first multicenter study including all official geographic regions of Brazil, with a significant number of women, of various socioeconomic stratifications and with evaluations of parameters related to menstruation according to the current worldwide accepted definitions. In addition, it is a study that allowed the comparison between subjective assessment (self-perception) of AUB and objective parameters that define the bleeding pattern, reinforcing the importance of valuing the woman’s complaint. As weaknesses, we emphasize that the etiological diagnosis of AUB, the history of hospitalizations and blood transfusions were obtained through self-reporting. Another weakness is the fact that women were included when receiving health care services. In Brazil, socioeconomic, geographical, and cultural discrepancies can hinder access to these services. Thus, the prevalence of AUB may be higher than that found by us.

The high prevalence of AUB and consequences such as anemia and worsening quality of life alert to the need to facilitate health care for these women. For women to be treated appropriately, it is essential that the problem be understood and known by women, health professionals and health managers. Although specific studies are needed, broad data, which provides more global health indicators, allows a larger assessment of the population’s needs, enabling public policy actions that improve health care and reduce risks and harm.

## Conclusion

The prevalence of AUB in Brazil is 31.4%, assessed by self-perception, in agreement with objective parameters that define AUB. Almost half of them have been diagnosed with anemia due to increased bleeding. About 50% of women of reproductive age report that their menstrual period has a negative impact on their quality of life, while the worsening occurs in 8 out of 10 women with self-perception of AUB.
